# miR-27-3p inhibition restore fibroblasts viability in diabetic wound by targeting NOVA1

**DOI:** 10.18632/aging.103266

**Published:** 2020-06-26

**Authors:** Peng Zhang, Xiaomei Song, Qirong Dong, Long Zhou, Lei Wang

**Affiliations:** 1 Department of Orthopedics, Suzhou Science and Technology Town Hospital, The Affiliated Suzhou Science and Technology Town Hospital of Nanjing Medical University, Suzhou 215000, China; 2 Department of Orthopedics, Second Affiliated Hospital of Soochow University, Suzhou 215000, China

**Keywords:** microRNAs, fibroblast, NOVA1, proliferation, migration

## Abstract

Diabetic wounds increase morbidity and decrease quality of life in patients with type 2 diabetes. Serum miR-27-3p levels are reportedly elevated in type 2 diabetic patients. In the present study, we explored the role of miR-27-3p during wound healing. We found that miR-27-3p is overexpressed in cutaneous fibroblasts of diabetic patients and mice. miR-27-3p knockdown enhanced the proliferation and migration of fibroblasts, while suppressing the incidence of fibroblast apoptosis. Overexpressing miR-27-3p in fibroblasts had the opposite effects. We also identified neuro-oncological ventral antigen 1 (NOVA1) as a target of miR-27-3p in fibroblasts. Knocking down NOVA1 using targeted siRNA mimicked the effects of miR-27-3p overexpression in fibroblasts. Administration of miR-27-3p to the area around wounds inflicted in mice delayed healing of those wounds. This suggests that miR-27-3p suppresses fibroblast function by targeting NOVA1, which results in the slowing of wound healing. These findings may offer a new approach to the treatment of diabetic wound healing.

## INTRODUCTION

Diabetic wounds impair the health and quality of life of patients with type 2 diabetes (T2D) and are associated with high mortality [1]. The incidence of diabetic wounds has been increasing in recent years, placing a heavy burden on healthcare systems [2]. A number of studies have focused on uncovering the pathological process underlying diabetic wounds, and progress has been made [3–5]. However, diabetic wound healing is a complex process affected by numerous factors, and our understanding remains incomplete.

miRNAs are small noncoding RNAs that mediate a wide range of biological processes by altering the expression of target genes [6]. During diabetic wound healing, evidence now indicates that miRNAs play crucial roles in mediating angiogenesis as well as cell proliferation, migration and apoptosis [7, 8]. It was recently found that high levels of miR-27-3p are present in serum from patients with T2D [9]. This upregulated miR-27-3p reportedly promotes both insulin resistance and diabetic retinopathy [10, 11], but its effect on diabetic wound healing and its underlying mechanism is still unclear. In the present study, therefore, we explored the effect of miR-27-3p on fibroblasts and its role in wound healing.

## RESULTS

### miR-27-3p is upregulated in fibroblasts in diabetic wounds

**Figure 1:**
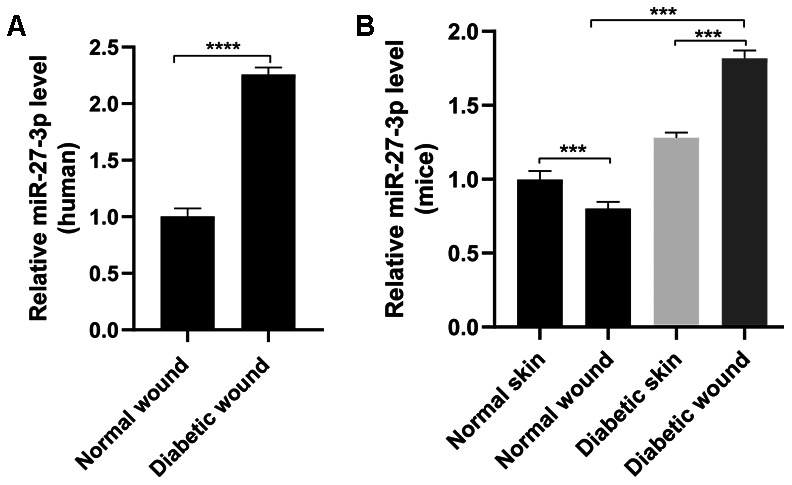
**miR-27-3p is upregulated in fibroblasts from diabetic wounds.** (**A**) miR-27-3p levels in fibroblasts from wounds in diabetic and otherwise healthy patients. (**B**) miR-27-3p level in fibroblasts from normal and wounds in diabetic and healthy mice.

To determine levels of miR-27-3p, we collected cutaneous tissue from diabetic and normal wounds, isolated the fibroblasts, and assessed levels of miR-27-3p expression using qRT-PCR. Our data showed that miR-27-3p expression was significantly higher in fibroblasts from diabetic wounds than normal wounds (Figure 1A). Likewise, miR-27-3p levels were significantly higher in skin fibroblasts from wounds in diabetic mice than healthy mice (Figure 1B).

### miR-27-3p impairs fibroblast function in vitro

**Figure 2:**
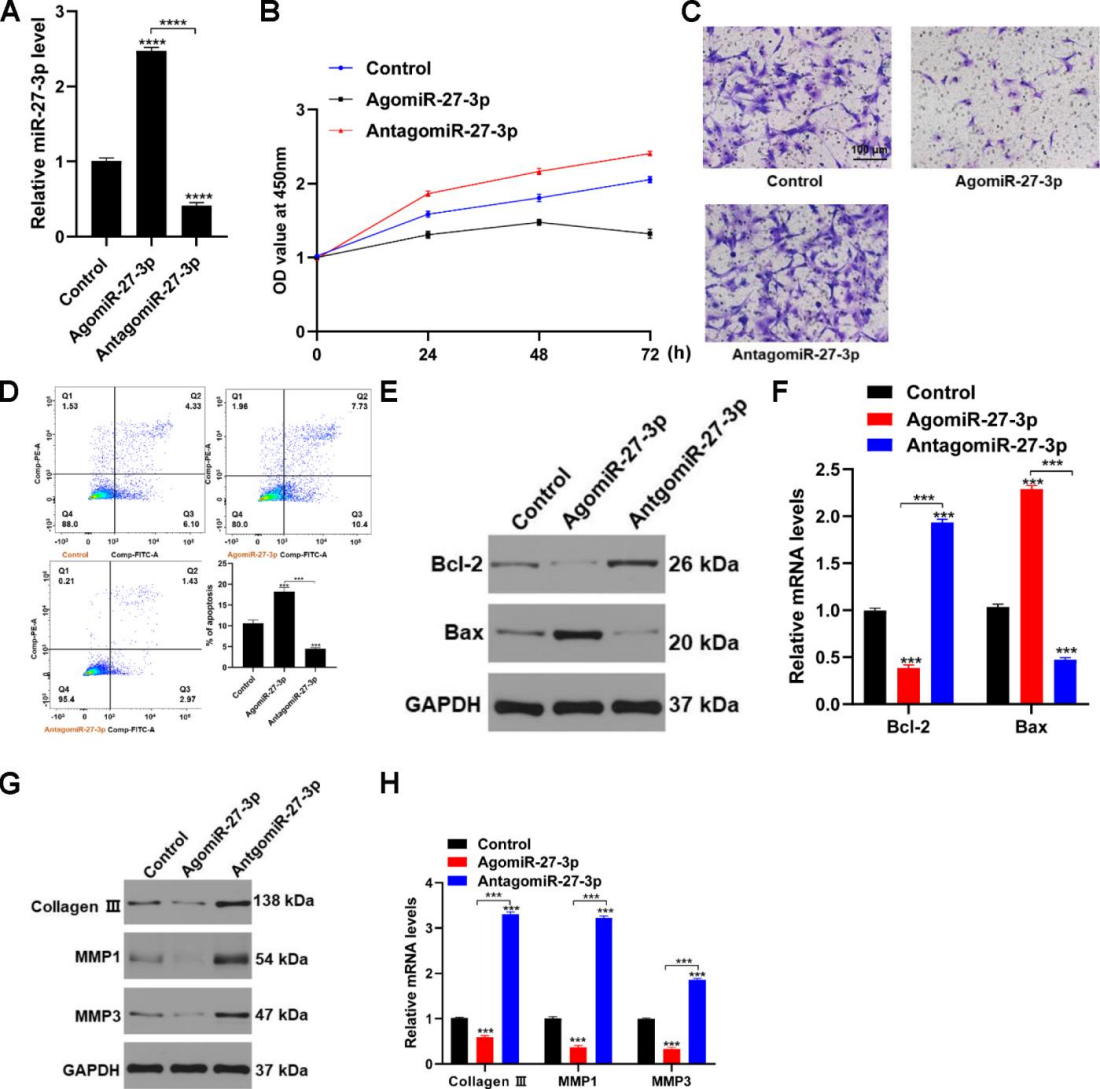
**miR-27-3p impairs fibroblast function in vitro.** (**A**) qRT-PCR was used to detected levels of miR-27-3p expression. (**B**) CCK8 assays were used to assess the viability of fibroblasts. (**C**) Fibroblast migration was evaluated using transwell assays. (**D**) Flow cytometry was used to evaluate apoptosis (Q2+Q3) among fibroblasts: Q1, dead cells; Q2, later apoptosis; Q3, early apoptosis; Q4, living cells. (**E**, **F**) pro-apoptotic and anti-apoptotic proteins were assessed with Western blotting and qRT-PCR. (**G**, **H**) The ECM-related proteins collagen III, MMP1 and MMP3 were evaluated with Western blotting and qRT-PCR.

To investigate the actions of miR-27-3p in fibroblasts, we transfected the cells with miR-27-3p mimic or inhibitor. Levels of miR-27-3p were significantly higher in the miR-27-3p mimic group than the miR-27-3p inhibitor group (Figure 2A). Transfecting fibroblasts with agomiR-27-3p inhibited proliferation, while miR-27-3p knockdown promoted fibroblast proliferation and migration (Figure 2B and 2C). In addition, miR-27-3p overexpression increased the incidence of apoptosis among fibroblasts as well as levels of the pro-apoptotic protein Bax (Figure 2D–2F). Exploration of the effects of miR-27-3p on expression genes related to extracellular matrix (ECM) revealed that miR-27-3p overexpression suppressed while miR-27-3p knockdown promoted expression of collagen III, MMP1 and MMP3 (Figure 2G and 2H). Thus, overexpression of miR-27-3p appears to impair fibroblast function.

### miR-27-3p affects fibroblast function by targeting NOVA1

**Figure 3:**
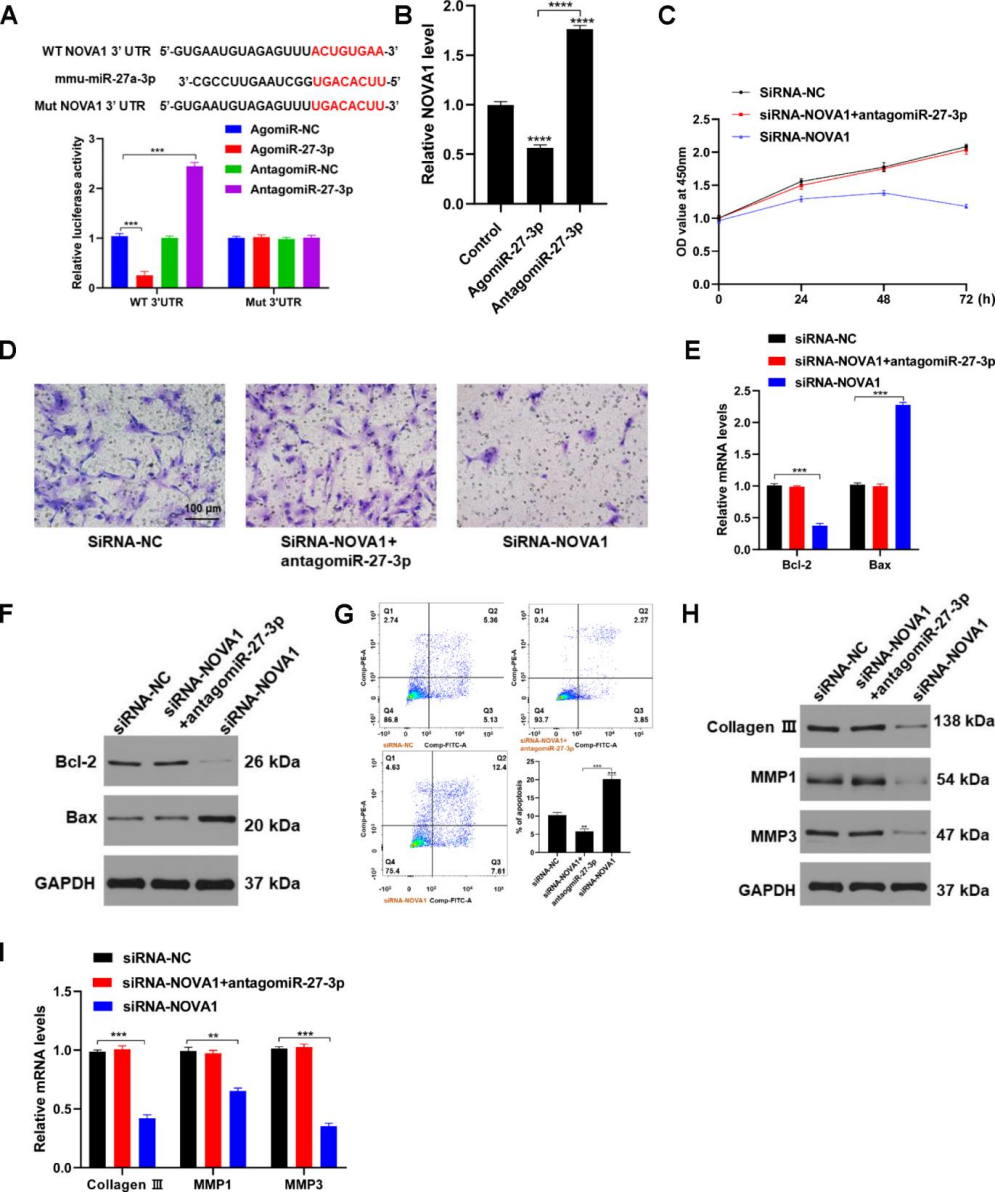
**NOVA1 is a novel target of miR-27-3p.** (**A**) The predicted miR-27-3p binding site within the NOVA1 3’-UTR was determined by Targetscan (Upper). miR-27-3p suppresses NOVA1 3’-UTR reporter activity (Lower). (**B**) qRT-PCR analysis showing that NOVA1 expression is suppressed in fibroblasts transfected with agomiR-27-3p. (**C**) CCK8 assays used to assess fibroblast proliferation. (**D**) Transwell assays were used to assess fibroblast migration capacity. (**E**, **F**) Expression of pro-apoptotic and anti-apoptotic proteins was detected with qRT-PCR and Western blotting. (**G**) Flow cytometry evaluating the cell cycle in fibroblasts. (**H**, **I**) The ECM-related proteins collagen III, MMP1 and MMP3 were evaluated with Western blotting and qRT-PCR.

Neuro-oncological ventral antigen 1(NOVA1) gene is predicted to be a target of miR-27-3p. Luciferase activity of the NOVA1 3’URT reporter was significantly suppressed by agomiR-27-3p but was enhanced by antagomiR-27-3p (Figure 3A). To determine whether fibroblast function is NOVA1-dependent, we assessed the effects of transfecting NOVA1 siRNA into fibroblasts (Figure 3B). The results showed that NOVA1 siRNA inhibited the both the proliferation and migration of fibroblasts (Figure 3C and 3D), and increased the incidence of apoptosis (Figure 3E and 3G). In addition, secretion of ECM-related proteins by fibroblasts was also suppressed by NOVA1 siRNA (Figure 3H and 3I). These results suggest that miR-27-3p directly targets NOVA1 in fibroblasts by interacting with its 3’-UTR.

### Wound healing potential of antagomiR-27-3p in vivo

**Figure 4:**
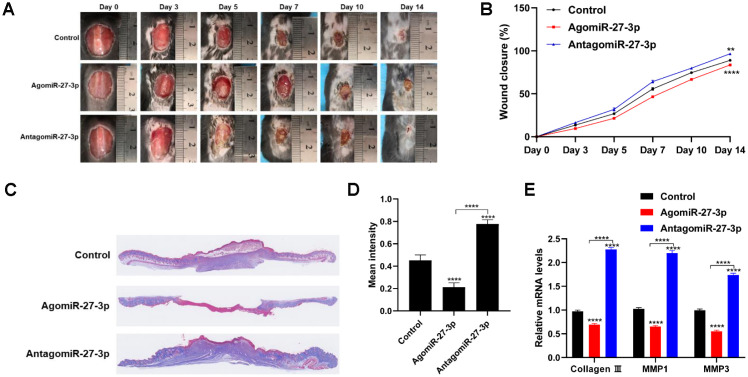
**Downregulation of miR-27-3p promotes wound healing in vivo.** (**A**) Digital photo of wounds treated with PBS, agomiR-27-3p, or antagomiR-27-3p. (**B**) Rate of wound-closure of the three groups. (**C**) Masson’s trichrome staining of wound sections treated with PBS, agomiR-27-3p, or antagomiR-27-3p. (**D**) Quantitative analysis of the mean intensity of Masson-stained areas in the three groups. (**E**) qRT-PCR evaluating the level of collagen 3, MMP1 and MMP3 expression. n=5. *p<0.05, **p<0.01, ***p<0.001.

To determine the role of miR-27-3p during wound healing, agomiR-27-3p and antagomiR-27-3p was injected around wound sites in mice. Fourteen days after wounding, mice administered antagomiR-27-3p showed more extensive healing than those administered agomiR-27-3p (Figure 4A and 4B). Consistent with the wound healing rates, histological analysis of Masson’s trichrome-stained sections showed agomiR-27-3p decreased amounts of wavy collagen fibers, while antagomiR-27-4p significantly increased the amounts of wavy collagen fibers (Figure 4C and 4D). In addition, miR-27-3p overexpression blunted expression of collagen 3, MMP1, and MMP3 (Figure 4E). These finding suggest that knocking down miR-27-3p promotes wound healing in vivo.

## DISCUSSION

In this study, we identified that miR-27-3p as a key mediator of fibroblast function. We showed that miR-27-3p is highly expressed in cutaneous fibroblasts from diabetic patients, and that miR-27-3p suppresses fibroblast proliferation and migration while increasing their incidence of apoptosis. We also confirmed that NOVA1 is a downstream target of miR-27-3p in fibroblasts, and that miR-27-3p delays wound healing in vivo.

Through post-transcriptional regulation of gene expression, miRNAs play key roles in a wide range of cellular processes and contribute to the pathogenesis of various diseases [12, 13]. During diabetic wound healing, for example, miRNAs exert effects at the inflammation, angiogenesis, re-epithelialization and remodeling stages [14]. Moreover, diabetes alters the expression of profile of miRNAs [15]. For instance, serum levels of miR-27-3p are significantly elevated T2D patients [9]. In the present study, our in vitro and in vivo observations indicate that miR-27-3p is also increased in fibroblasts from diabetic patients and mice, and that miR-27-3p may delay wound healing by impairing fibroblast functionality.

NOVA1 is a neuron-specific pre-mRNA binding splicing factor, which is highly expressed in tumor cells [16]. NOVA1 knockdown effectively suppresses tumor growth [17]. Recent studies also showed that NOVA1 is enriched in normal fibroblasts [18]. Because fibroblasts play vital roles during wound healing, we investigated whether NOVA1 knockdown could affect the function of cutaneous fibroblasts. Our data suggests that NOVA1 knockdown impairs fibroblasts function, as evidenced by suppressed fibroblast proliferation and their enhanced apoptosis. Moreover, NOVA1 expression is suppressed by miR-27-3p agonist. Thus, NOVA1 appears to be essential for fibroblast function, and its activity is negatively regulated by miR-27-3p.

Although our study indicates the negative role of miR-27-3p during wound healing, it should be noted that numerous miRNAs are involved in the process of wound healing, and many of those play dual roles during wound healing. Moreover, our study explored only the effect of miR-27-3p on fibroblasts, it may affect the process of wound healing by exerting effects on vascular endothelial cells and keratinocytes.

Taken together, our findings are compelling evidence that miR-27-3p is a novel regulator of fibroblast function, which acts at lease in part via NOVA1. In addition, we show for the first time that miR-27-3p activity impairs the process of wound healing. These findings may provide new perspective for the treatment of wound healing.

## MATERIALS AND METHODS

### Sample collection

Skin samples from diabetic and normal wounds were collected from 10 patients (5 diabetic and 5 healthy) in the Department of Orthopedics, Second Affiliated Hospital of Soochow University. The clinical characteristics of those patients are shown in Supplementary Table 1. The average age of volunteers was 66.6 ± 8.82, 60.6 ± 5.07 in healthy group and diabetic group, respectively. The Committees of Clinical Ethics at the Second Affiliated Hospital of Soochow University (Souzhou, China) approved all studies, and informed consent was obtained from all participants. Skin samples from diabetic and healthy wounds or normal skins were also collected from diabetic and wide-type C57/BL6 mice. All animal studies were approved by The Institutional Animal Care and Use Committee at the Second Affiliated Hospital of Soochow University (#No. 2019-372-33).

### Cell culture and transfection

Fibroblasts were purchased from the Cell Bank of Wuhan University, Wuhan, China. The cells were cultured in DMEM/F-12 (#10565042, ThermoFisher Scientific) supplemented with 10% fetal bovine serum (FBS) at 37°C under 5% CO_2_. Cells were transfected with 200 μm agomiR-27-3p, antagomiR-27-3p or 50 nm of NOVA1 siRNA using Lipofectamine 3000 (L3000001, Thermo Fisher Scientific, USA).

### Cell counting kit 8 (CCK8) assay

After fibroblasts were seeded into 96-well plates and treated by agomiR-27-3p, antagomiR-27-3p, or NOVA1 siRNA for 24, 48 or 72 h, 10 ul CCK8 reagent was added to the medium for 2 h. Cell viability was evaluated by measuring absorbance at 450 nm.

### Cell migration

Transwell chambers were used to measure cell migration. Briefly, 5 x 10^3^ cells were seeded into the upper charmer, and medium containing 10% FBS was added to the lower charmer. After incubation for 24 h, the migrated cells were stained with crystal violet and observed under an optical microscope (IX51, Olympus, Tokyo, Japan).

### Flow cytometry

After cells were incubated with indicated treatment for 24 h, cells were collected for further analysis. Cell apoptosis was evaluated using the Annexin V-FITC-PI apoptosis detection kits (GeneChem, Shanghai, China) according to the manufacturers’ instruction (BD AriaIII, New Jersey, BD, USA)

### qRT-PCR

Skin wound tissues or fibroblasts were collected to analysis the mRNA gene expression. Briefly, otal RNA was isolated using a RNeasy Mini Kit (QIAGEN, Germany) and reverse-transcribed into complementary DNA. qRT-PCR was performed using the StepOneTM Real-Time PCR (Life Technologies, Carlsbad, CA, USA). The primer sequences are listed in Table 1. The relative expression levels were calculated using the ΔΔCT method and normalized to the level of GAPDH expression, which served as an internal control.

**Table 1:** The primer sequence of genes.

**Gene**	**Primer (5’ to 3’)**
MMP1 Forward	GGCTGAAAGTGACTGGGAAACC
MMP1 Reverse	TGCTCTTGGCAAATCTGGCGTG
MMP 3 Forward	CTGGACTCCGACACTCTGGA
MMP 3 Reverse	CAGGAAAGGTTCTGAAGTGACC
COL3A1 Forward	GCCAAATATGTGTCTGTGACTCA
CLO3A1 Reverse	GGGCGAGTAGGAGCAGTTG
GAPDH Forward	GGCACAGTCAAGGCTGAGAATG
GAPDH Reverse	ATGGTGGTGAAGACGCCAGTA

### Western blot

Wound tissue and fibroblasts were lysed in lysis buffer, after which protein levels in the lysate were measured using a BCA protein assay kit. Equal amounts (40 uL)of total protein were resolved on 10% SDS-PAGE, then transferred to polyvinylidene difluoride membranes. The membranes were incubated first with primary antibodies at 4°C overnight and then with the HRP-conjugated secondary antibodies for 1 h at 37°C. Band visualization was carried out with an ECL Advance Western Blotting Detection Kit. Densities were calculated by the Quantity One Software (Canon, Melville, NY) and normalized by GADPH. The primary antibodies used were as follow: anti-Cyclin D1 (1:1000, Abcam, MA, USA), anti-Cyclin D3 (1:1,000, CST, USA), anti-Bcl-2 (1:2000, Abcam, MA, USA), anti-Bax (1:2000, CST, USA), anti-GAPDH (1:10,000, Abcam, USA).

### Mouse wound model

C57/BL6 mice (6-8 weeks old) were purchased from The Center of Experimental Animals, Soochow university. After mice were anesthetized with sodium pentobarbital (30 mg/kg), the back hairs were shaved and full-thickness dorsal wounds (10 x 10 mm^2^) were made. The animals were then randomly divided into three groups, which were administered equal amounts (100 μL) of PBS, agomiR-27-3p, or antagomiR-27-3p were injected around the wound (25 μL/site, 4 sites). Mice were sacrificed for further analysis on day 14 after wounding and wound samples were collected.

### Masson’s trichrome staining

Collected wound samples were fixed in paraformaldehyde (4%, pH 7.4), and then embedded in paraffin and cut into 4-μm-thick sections, which were stained with Masson’s trichrome to assess the degree of collagen maturity.

### Statistical analysis

All data are shown as the mean ± SD. Statistical analysis was performed using Graphpad Prime 8.0. Student’s t test and one-way ANOVA with Dunnett’s post hoc test were applied as appropriate to make comparisons between two or multiple groups, respectively. Values of P<0.05 were considered significant.

## Supplementary Material

Supplementary Tables
