# Supplementary material for: miR-27-3p inhibition restore fibroblasts viability in diabetic wound by targeting NOVA1
Source: Aging (Albany NY). 2020 Jun 26;12(13):12841–9. doi: 10.18632/aging.103266 (PMC7377889; doi:10.18632/aging.103266)
Supplement: Supplementary Tables [file aging-12-103266-s001..pdf]

SUPPLEMENTARY TABLE

Supplementary Table 1. The clinical information of patients.

Number	Sex	Age	Wound site	Healthy status
1	Male	76	Left foot	Healthy
2	Female	53	Left foot	Healthy
3	Female	64	Right foot	Healthy
4	Male	68	Left foot	Healthy
5	Female	72	Right foot	Healthy
6	Female	54	Right foot	Diabetes
7	Female	59	Right foot	Diabetes
8	Male	62	Left foot	Diabetes
9	Male	60	Right foot	Diabetes
10	Female	68	Left foot	Diabetes
